# Immigrant women living with HIV in Spain: a qualitative approach to encourage medical follow-up

**DOI:** 10.1186/1471-2458-14-1115

**Published:** 2014-10-29

**Authors:** Anne Guionnet, Bárbara Navaza, Belén Pizarro de la Fuente, María Jesús Pérez-Elías, Fernando Dronda, Rogelio López-Vélez, José A Pérez-Molina

**Affiliations:** Tropical Medicine, Infectious Diseases Department, Hospital Universitario Ramón y Cajal, IRYCIS, Crta. de Colmenar Km 9,1, Madrid, 2834 Spain; Infectious Diseases Department, Hospital Universitario Ramón y Cajal, IRYCIS, Madrid, Spain

**Keywords:** Women, HIV, Migration, Lost to follow-up, Barriers, Facilitators

## Abstract

**Background:**

Immigrant women living with HIV generally have worse adherence to medical treatment and follow-up when compared to native women and immigrant or native men. The general aim of this study was to improve healthcare services for HIV-positive women and to better understand why some of them discontinue treatment. The specific objectives were: (1) to explore the barriers and facilitators to medical follow-up among women and (2) to use the findings to create a guide for healthcare professionals with strategies and tools to encourage the immigrant women to continue with their healthcare treatment.

**Methods:**

We conducted a qualitative, patient-centred research based on semi-structured interviews in order to understand the drivers and barriers for HIV positive immigrant women to adhere to medical follow-up. A total of 26 women in active or discontinued treatment (from sub-Saharan Africa (10), Latin America (8) and Spain (8)) were interviewed in 2012 using a purposive sampling methodology. The semi-structured interviews were transcribed and analysed based on the grounded theory approach and the framework method. Three researchers took part in the triangulation of results.

The study was approved by the Ethical Committee of the Hospital Universitario Ramón y Cajal.

**Results:**

The study revealed eight categories that impacted adherence to treatment and medical follow-up: doctor-patient relationship, relationship between body and HIV, employment, gender roles, representations of AIDS, emotional support received, trust in biomedical system, and psychological condition. Specific barriers and facilitators related to these categories were identified. In immigrant women, the influence of these barriers was greater than in Spanish women.

Recommendations for healthcare professionals based on this study have been compiled in an informative brochure.

**Conclusions:**

Social, cultural, and psychological aspects as well as self-perception of body changes, gender roles, and the relationship with the healthcare system, are key elements that may affect the adherence to medical treatment of immigrant women living with HIV.

Qualitative research focused on the comprehensive experience of living with HIV can be useful for creating tools that pave the way to detect barriers and facilitators to medical follow-up in specific populations.

## Background

Women are estimated to make up half of the total HIV infected population worldwide. Although the gender distribution varies depending on the location, epidemiological data demonstrate that in recent years the proportion of infected women is growing [1]. Compared to men, women represent a more vulnerable population because they are more frequently exposed to discrimination, gender violence and overall infringements of basic rights [2, 3].

Furthermore, it is known that the effect of the antiretroviral treatment can vary according to gender. Although the study results obtained on women are contradictory and vary from better responses [4–6], similar [7, 8] or worse [9, 10] than those among men, what has been consistently demonstrated by studies is that the ratio of discontinued antiretroviral treatments is significantly higher among women [7–10], especially in the case of immigrant women [11]. HIV infected immigrants are an especially vulnerable population, particularly immigrants who are women, due to cultural barriers, social stigma associated with the disease, difficulties in accessing health care, and language barriers [12–14]. In a qualitative study focused on HIV positive Latin American immigrants living in the US, the authors identified two main barriers to obtaining the correct treatment for the disease: (1) being undocumented and (2) the stigma associated with the virus both in the family and in society at large [14]. In the United Kingdom, a study revealed that sub-Saharan patients were taking traditional medicines to treat HIV without informing their doctors, showing the influence of cultural conceptions in regards to HIV treatment [15]. Another of the investigations focusing on HIV positive immigrant women showed that religion, the migration process and the stigma associated with AIDS significantly influenced how they dealt with the infection [16]. The language gap has also been described as a factor that influences their vulnerability to the HIV [17] and campaigns aimed at preventing and treating the disease are often not adequate for immigrant women because of their language constraints.

In Spain, more than half of the women diagnosed with HIV in recent years are immigrants and up to 30% of the AIDS cases among women are found among Latin American and sub-Saharan immigrants [18]. Existing data shows that the rate of medical loss to follow-up among immigrant women can be up to twice that of Spanish women [19], a situation that may worsen with the present economic crisis [20]. Nevertheless, there is lack of evidence able to explain the reasons why this population group has a worse adherence to HIV treatment and follow-up. Even if in another countries research has shown the importance of factors such as stigma, religious beliefs or migration status, in Spain no qualitative study has been developed yet to investigate the causes why immigrant women stop HIV treatment. The aim of this paper is twofold: Firstly, to describe the barriers and facilitators of social, cultural, and psychological origin that affect the medical management and follow-up treatment of HIV positive immigrant women in Spain. Secondly, to identify new strategies and tools for healthcare professionals to assist them in encouraging follow-up treatment among this population.

## Methods

This qualitative study was developed by the Infectious Diseases Department of the Hospital Ramón y Cajal in Madrid between January and July 2012. The qualitative approach was deemed appropriate as it is well suited for the difficult task of representing groups outside the society's mainstream as well as for providing rich descriptions of complex phenomena.

### Sample

Both immigrant and native women infected with sexually-transmitted HIV were selected using a purposive sampling method in order to facilitate the identification and selection of information-rich cases; the blood transmission of HIV was an exclusion criterion for the participation in the study as it was not a major way of transmission among the immigrant population.

The women were selected on the basis of two criteria. Firstly, country of origin; a distinction between immigrant and native women was made in order to investigate factors that were specific to migrant women leading them to stop treatment. This study focused on first generation sub-Saharan and Latin American immigrants in Spain, due to their prevalence of HIV infection [18]. The second criterion was adherence, in order to find the barriers and facilitators to HIV medical follow-up, a comparison was made between women who interrupted the treatment and those who had a correct adherence. We used a set of determining factors in distinguishing those women who had poor adherence, namely not attending at least two appointments at the clinic (Infectious Diseases Consultation. Hospital Ramón y Cajal) within six months and not providing reason.

Participants from different educational backgrounds (primary/secondary/graduate studies), ages, marital status (single/married), number of children and employment status (employed/unemployed) were included in order to cover a wide range of the general population. The number of women included in the study was determined by theoretical saturation [21], that is to say, until there were no new information for defining categories or the relationships among them.

All the women who had stopped treatment and were reachable accepted to participate in the research without economic incentive.

Nonetheless, 8 immigrant women with HIV who had stopped medical follow-up in the consultation could not be contacted through the telephone or address registered in the hospital.

### Data collection

The patients were contacted and invited for an interview at a time and place of their choice (hospital, residence, or other preferred venue). Patients were first contacted by their personal physicians, who asked for their consent to participate in the study. Afterwards, they were contacted by the interviewer who arranged the meetings. Thus, six physicians (three men and three women) referred their own patients to participate in the research.

Semi-structured interviews were carried out in Spanish, French and English and the mean duration of the meetings was one hour. A bilingual (French/Spanish) psychologist with experience in the follow-up of HIV positive patients conducted the interviews. A professional interpreter also participated in the interviews with English-speaking patients. Patients were informed about the aim of the research: to improve healthcare services for HIV-positive women and to better understand why some of them discontinue treatment. All women and investigators signed an informed consent form, approved by the Ethical Committee of the Ramón y Cajal Hospital, in order to assure the confidentiality to the patients. The interview script permitted participants to explore the following dimensions: a) their own experience of the disease; b) social and family network, c) HIV and AIDS representations in the country of origin and in the host country, d) the migration process, e) relationship and communication with the healthcare system and healthcare professionals, f) Motivations and constraints on adherence to treatment. The interviews were recorded and transcribed for the analysis.

### Analysis

The analysis was based on the grounded theory approach introduced by Glaser and Strauss [21, 22]. This method was chosen because we wanted to investigate in an inductive way the categories that influence lost-to-follow up among immigrant women. Researchers read (and re-read) the corpus of data (transcripts of interviews) and label categories and their interrelationships. The framework approach analysis was used in order to systematically index and chart emerging topics related to the dimensions explored during the interviews, to facilitate interactive comparisons across cases and examine relationships between categories [23].

In order to cross-validate the results (triangulation), multiple data sources were analysed. Thus, we included in the analysis the perspective of native women and also of women with a good adherence to treatment, in addition to immigrant women and women los-to-follow up. Another triangulation strategy was including three researchers in the analysis process (a doctor and two psychologists). These researchers (A.G., B.P.F., J.A.M.) held four meetings where they discussed their individuals findings and provided different perspectives of the global results with no significant disagreements. Finally, results were presented to the participants in individual meetings to receive their feedback.

## Results

Twenty-six women were interviewed: 8 from Latin America, 10 from sub-Saharan Africa and 8 Native to Spain. The median age was 41 years old, 23.1% had only attended primary education, 61.5% had no partner and only 50% of them were employed. Six of them (23.1%) had stopped treatment (Table 1).

**Table 1:** **Sociodemographic characteristics of the studied population**

Region of origin	Country of origin	Age (years)	Education level	Partner (Yes/no), children	Working (Yes/No)	Interruption follow-up
LA1	Equator	25	Secondary	No, 1	No	Yes
LA2	Venezuela	30	Secondary	No, 0	No	Yes
LA3	Dominican Republic	39	Secondary	No, 1	Yes	No
LA4	Dominican Republic	40	University	No, 1	Yes	No
AL5	Paraguay	44	Primary	No, 2	Yes	No
LA6	Equator	33	Secondary	No, 1	Yes	No
LA7	Dominican Republic	56	Primary	No, 3	Yes	No
LA8	Bolivia	32	Secondary	Yes, 1	Yes	No
SA1	Cameroon	36	Primary	Yes, 2	No	No
SA2	Equatorial Guinea	70	University	No, 5	No	No
SA3	Equatorial Guinea	28	Secondary	No, 1	No	No
SA4	Equatorial Guinea	43	Secondary	Yes, 2	No	No
SA5	Nigeria	34	Secondary	No, 0	Yes	Yes
SA6	Equatorial Guinea	32	Primary	Yes, 2	No	No
SA7	Equatorial Guinea	46	Secondary	No, 3	Yes	Yes
SA8	Guinea Conakry	26	University	No, 2	No	No
SA9	Equatorial Guinea	37	Secondary	No, 0	Yes	Yes
SA10	Equatorial Guinea	42	Secondary	Yes, 1	No	No
S1	Spain	55	University	Yes, 1	No	No
S2	Spain	36	University	No, 0	No	No
S3	Spain	48	Secondary	No, 1	Yes	Yes
S4	Spain	49	Secondary	Yes, 0	No	No
S5	Spain	54	Secondary	No, 3	No	No
S6	Spain	47	Primary	Yes, 2	Yes	No
S7	Spain	44	Secondary	Yes, 1	Yes	No
S8	Spain	48	Primary	Yes, 1	Yes	No

Elements that were hampering, preventing or easing the women's follow-up treatment emerged throughout the analysis and were grouped in the following categories: 1) doctor-patient relationship, 2) relationship between body and HIV, 3) employment, 4) gender roles, 5) representations of AIDS, 6) emotional support received, 7) trust in biomedical system and 8) psychological condition.

We did not find any categories specific for the immigrant women compared to the native women, thus facilitators and barriers mentioned for each category apply to both groups. Nevertheless, we found specific features regarding immigrant women which are explained in the subtitle “Immigrant women”.

### Doctor-patient relationship

The quality of the doctor-patient relationship greatly impacted the likelihood of adherence to treatment. We found some facilitators and barriers regarding this relation.

#### Facilitators

Women expressed that a good relationship with their medical practitioner was crucial to follow up treatment. For many of the immigrant and native women, the medical consultation was the only place where they felt comfortable enough to talk about the disease, which led to certain expectations regarding medical professionals. In their opinion, doctors should:

1. Serve as experts. The practitioner is expected to provide information about HIV, the ways of transmission, the latest research activities and treatments. This information provided the patients with a better understanding of the disease and decreased their HIV-related anxiety.

*“It was very shocking and when I was told, I went to a private doctor, I paid him, we sat down, he informed me well, very well, of everything, so the information he gave me helped me to keep calm, because I had my ideas about what it is to have HIV”. (SA6, Equatorial Guinea)*

2) Provide emotional support: Women had a very positive view of the emotional support that doctors could offer when they were feeling depressed. They expected doctors to know how to gently break the news and to be more closely involved in their lives by asking questions such as: “How are you?” “How are things going?” They also appreciated positive comments about their body condition, especially when they were feeling fragile, because it helped to improve their self-esteem.

*“The doctor gives me a lot of support, he tells me: ‘You are great, splendid, you are very pretty’ ”. (LA8, Bolivia)*

3) Serve as an interpreter: the doctor should translate the scientific HIV-related language so as to be understandable and therefore let the patient feel more in control of the disease.

*“What one needs is some support, to feel the doctor close to you and to receive the information in a way that one is able to understand”. (S1, Spain)*

Most of the interviewees showed no preference for male or female doctors, only two immigrant women said they felt more comfortable with a woman practitioner.

#### Barriers

Conversely, a bad relationship acted as a barrier. A bad relationship could result from the feeling of being judged by the healthcare professional.

*“I stopped the follow-up, I didn’t feel like going to the doctor and I got very angry at the doctor there, I didn’t like the way he treated me…he treated me badly, I felt humiliated, I didn’t want to go to doctors at that time, plus the way the doctor treated me, I stopped going....6 years....and I was all right." (S3, Spanish)*

#### Immigrant women

Immigrant women explained that they had a relationship of great inequality with their doctors. For them, doctors represented authority and thus commanded respect, so patients felt that they could not question their recommendations. On the other hand, native women described having a relationship of greater equality with health practitioners: They found no constraints to ask questions if they did not understand something and they could more easily express their needs for emotional support.

Regarding women from sub-Saharan Africa who spoke only English or French, the language gap was an important barrier.

### Relationship between body and HIV

The different stages of HIV infection and how the body reacts in each of them can affect medical care.

#### Facilitators

Feeling the disease in the body (having symptoms) makes the compliance with follow-up treatment easier to achieve. Those women, who learned they had HIV when they already had AIDS and consequently experienced an improvement in their health with the treatment, said this was an encouraging factor in continuing the treatment.

Doctors are the only ones that can inform patients about the state of their health during the HIV asymptomatic stage. This gives doctors an important power and reinforces the need for a good relationship.

#### Barriers

The women expressed the conflict of having a serious disease and not feeling any bodily symptoms. A woman from Equatorial Guinea who was going through the asymptomatic stage told us how her anxiety led her to feel what seemed to be HIV symptoms; as a consequence, she kept a constant relationship with the healthcare staff.

*“Sometimes I feel dizzy and I feel like I’m having an attack and after five minutes, it stops and I start trembling, that is how I got to know HIV symptoms”. (SA3, Equatorial Guinea)*

Diseases are usually perceived and described through symptoms and reactions of the body. Coming to terms with an HIV infection, which may show no symptoms, may be difficult. When HIV is asymptomatic, it can be an obstacle to staying on track with follow-up treatment. All of the women who had interrupted treatment explained that they “were feeling fine” when they stopped.

#### Immigrant women

Immigrant women expressed the need to feel some symptoms in their body in order to experience the disease.

*“Those who think AIDS does not exist, won’t go to the doctor until they have symptoms”. (SA1, Cameroon)*

### Employment

Working and having a chronic disease with a need of a regular medical follow-up implies a specific management of the situation.

#### Facilitators

Women appreciated physicians who were flexible and allowed them to reschedule appointments, or even gave them the results over the phone, if necessary.

*“My doctor helps me a lot. He schedules all my appointments for the same day, he does it in the office, and when for some reason I cannot go, he calls me and asks “What happened? Couldn’t you come today?”” (SA9, Equatorial Guinea)*

#### Barriers

Hospital consultations can take time which makes it difficult for the patient to estimate how long she will be absent.

*“It is hard to come to the medical appointments because of work. It’s a hard job, I spend all day with an elderly woman…. […] I cannot be out of work, my daughter has a lot of expenses and I do not want her to say ‘listen, I don’t have a profession because my mother…’” (LA3, Dominican Republic)*

Although confidentiality is legally protected, there are employers who pressure their employees to explain their need for sick leave.

*“People say that no one has to know, that you don't have to tell why you are going to the doctor but that is not real, when you have a job and you constantly need to go to the doctor. You have to be absent many times and to lie a lot. And if you are a good liar, everything is alright but if you are bad at it, you do not believe your own lie and then they will notice something is wrong". (S4, Spain)*

Another barrier arises from the accessibility of the Spanish healthcare system and the centralisation of HIV positive patients in hospital consultations. The time allotted for these consultations is quite rigid and the medication is only provided for free in hospital pharmacies. As a result, patients are forced to go to the hospital during working hours and sometimes more than once, if their medical appointment is not scheduled on the same day that their medicines are available.

#### Immigrant women

The motivations for migration (often related to economic hardship or healthcare needs) can be an important barrier or facilitator regarding medical follow-up. Women who migrate for economic reasons expressed the vital importance of working, not only for them but also for their families in the country of origin. Work for the sake of the family takes priority over their own health. All of the immigrant women who had a job were working in the elderly care field or in cleaning services (for companies or private homes). Usually, this kind of work has inflexible shifts that leave little time for personal matters.

Inversely, when the migration is motivated by healthcare reasons, the woman feels she has the right, if not the moral obligation towards her family (that contributed financially to the trip) to regularly attend medical consultations and adhere to treatment.

*“My family, they sent me here to be treated. My sister told me that to be infected by the HIV is not as if you were going to die right now, it’s a disease that will require you to take pills, they will control it at the hospital, and you cannot stop going to the doctor." (SA3, Equatorial Guinea)*

Immigrant women have developed their own strategies for meeting both their employment duties and their medical follow-up. For example, they may ask a family member to pick up the medicine or ask a work colleague with a higher-level position to help them justify their absences. Workers with more flexible conditions were able to take free days to attend medical consultations.

### Gender roles

Being a woman often implies being a caretaker, being faithful and bearing children. Tradition-bound gender roles can appear in women's discourse and can act as barriers or facilitators for the adherence to medical treatment.

#### Facilitators

Gender roles could reinforce the willingness to take care of themselves and continue treatment in those women who were already performing functions of mothers and wives before becoming aware of their infection. The main motivations to go to the hospital were their children or husbands. The discourse was: “I take care of myself to take care of my family”.

*“I was motivated by my daughter who was very little. The need to protect her and put her first called for me to be well.” (S5, Spain)*

#### Barriers

Nevertheless, gender roles act as barriers when they are not fulfilled, for example, among younger women with no children or no stable partner. Thus, one young woman told us that she would not come to her scheduled appointments in case she was with her boyfriend so as to avoid explaining her visits to the hospital.

*“I don’t go to the hospital because sometimes my partner is with me and what should I say? ‘I have to go to the hospital’… and for what? I have to make excuses so I prefer not to go and save myself the trouble.” (LA1, Equator)*

#### Immigrant women

The influence of gender roles depends on the culture and the family in which the woman grew up; among immigrant women, gender roles were more pronounced. Only among a few Spanish women did we detect a different discourse that valued women according to criteria other than caretaking and reproduction.

### Social representations of AIDS

The values, ideas and beliefs that women shared about HIV and AIDS have an impact on the medical follow-up.

#### Facilitators

Women said that the information doctors provide their patients can change the perception of HIV as a death sentence, or as a disease only found among marginalised groups. By explaining that HIV is a chronic disease and not necessarily a death sentence doctors can encourage the patient to thrive and make plans for the future.

*“First, I thought: ‘Oh, my God, now I cannot do anything of my life.’ I remember once I told the doctor that instead of coming to Spain, my dream was to build my own house in my country. She told me that nothing was wrong, that I could do it, that everything will be alright with me. And I did it. Now I fell like a real person.” (SA10, Equatorial Guinea)*

#### Barriers

Because of its relationship to sexuality and blood, women refer to HIV and AIDS as a taboo disease, reinforcing its association with marginal groups. These perceptions of AIDS as a disease of “sluts” and “junkies” can hinder the disclosure of the infection to significant others and encourage denial of the disease. The fear of being associated with marginalised groups varied according to the origin of the women: Spanish women feared being associated with drug addicts, and immigrant women, with prostitutes or promiscuous behaviour.

*“In Guinea it has a bad reputation, if they say you have the disease, you're the sluttiest of all or you sleep with anyone, that is how they see it, that's how it is.” (SA10, Equatorial Guinea)*

*“If I say it, they would be talking, it’s associated with drugs, I don't want them to think I'm a drug addict.” (S3, Spain)*

Because of the gap between their self-perception (I’m not a prostitute, I’m not a junkie) and the social perception of HIV (HIV equals marginalised groups), some women felt compelled to repeat the tests in several hospitals without informing the medical staff.

We identify three reasons why the women wouldn't disclose their HIV status, which were closely linked to the social representations of HIV and AIDS.

1. The fear of rejection and social death. African women explained that family members controlled important relationships. For example, when two people fall in love, the families investigate the reputation of suitor. If they discover that the suitor has HIV, the marriage will be denied. The women were also very afraid that no one would attend their funeral if they die of AIDS. This shows that the social stigma attached to the disease goes beyond death.

A woman’s partner did not always play a supportive role; there were women who decided not to disclose their infection because they were afraid they would be accused of bringing the disease to the family. We interviewed victims of gender violence who could not negotiate having protected sex with their partners or women who were in a marriage of convenience, and even though they knew their husband had given them HIV, they never talked about it.

2) The fear of being labelled. Women preferred not to tell people they’ve just met that they have HIV, to avoid bias in the encounter.

When going to the hospital, they also tried to keep it confidential and were scared of running into acquaintances.

3) Fear of hurting loved ones. This is especially relevant for not disclosing the HIV diagnosis to parents and offspring (even if they are adults).

#### Immigrant women

AIDS as an equivalent of death is the perception of most immigrant women, since the access to treatment and medical attention depends on the economic situation of the person. The majority of the immigrant participants came from low-income families and had migrated to improve their living standards and those of their family.

*“I think it’s very bad, very bad, very bad, I think I will die or something […] the physician, I’m not sure if he told me the truth or if it’s just to ensure I don't have fear […] because if I have to die, I’d like to die in my country instead of here.” (SA7, Equatorial Guinea)*

The discourse from the Spanish women infected in the beginnings of the pandemic reveals a change in the perception of AIDS. They spoke of a time when people would die from HIV and that now it is simply a chronic disease, thanks to antiretroviral drugs.

*“To me, HIV is a pill that I take at night and that’s it. I also take a pill for cholesterol when I have it a little above average, but I never felt sick.” (S8, Spanish)*

Immigrant women had developed their own strategies for keeping their HIV infection confidential: They looked for hospitals far from where they lived; they asked for the last appointment available in the day, to avoid being seen by other patients; some of them preferred to wait outside of the consultation room and asked the doctors to call them on their mobiles when it was their turn.

Immigrant women thought that being treated in Spain rather than in their countries of origin was a facilitator to getting treatment. They explained that in their countries of origin, hospitals would meet with HIV positive patients in a separate building from the main hospital, thus making it difficult to maintain confidentiality.

Similarly, they felt relieved when medical consultations were not exclusively for HIV patients, making it more difficult to know why people are there.

*“While you are waiting here [in the tropical medicine unit], people ask you: ‘Dear, why are you here?’ I always answer that it is because I have Chagas [Endemic disease from Latin America transmitted by a triatomine bug].” (LA8, Bolivia)*

### Emotional support received

#### Facilitators

Having someone who provides emotional support is a facilitator for the medical follow-up. This person can be present at the consultations and can become the doctor’s ally, by faithfully repeating the recommendations and medical explanations. These people can be very important, as some women who had stopped treatment said they resumed it because someone close and important to them had encouraged them to go back to medical consultation.

*“When they told me here that I had HIV, my sister was with me in the consultation. I was feeling very bad, very bad, very bad, I started crying. But then, as my sister was here, she told me that having HIV does not mean that I was going to die because there was medication, the doctor was going to treat me…” (SA3, Equatorial Guinea)*

#### Barriers

Not to disclose their HIV status prevented women from finding emotional support in their family and social networks. They explained how difficult was for them to manage this chronic disease on their own for a long time. They also related periods of depressions and willingness to give up medical treatment due to this lack of emotional support. All the women who abandon the follow up did not disclose their HIV status with their relatives.

*“I was in another world, I mean, I didn’t […] because having it means a quite strong psychological thing and there are moments when you want to be away from it, I don't know.” (LA2, Venezuela)*

The family support is not as clear as in other diseases; women sometimes thought that by sharing their condition with their relatives the effect would be more devastating than if they kept it a secret.

*“My family, if they find out, they going to cold-shoulder me, that is my fear…I prefer not to tell them because I know they are going to humiliate me, then I prefer not to say anything.” (LA8, Bolivia)*

#### Immigrant women

Being an immigrant usually implies being apart from their families. Thus, many immigrant women won't have the possibility of receiving family support to help them face the disease.

**Figure 1:**
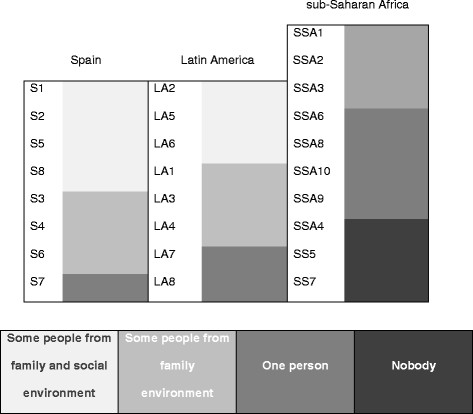
**Diagnosis disclosure according to region of origin.**

We observed that among African women the impact of fear created an evident isolation with no possibility of sharing their illness with people close to them (Figure 1).

When they chose someone to give them support, it was because that person knew how to keep a secret, had training in the healthcare field, had previous contact with HIV people, or did not judge them.

### Trust in biomedical system

Sometimes women showed a lack of trust on the biomedical system and therefore, on antiretroviral treatment.

#### Barriers

There were some doubts about the benefit of the treatment and the benevolent role of the doctors. These ideas were linked to the doubt of the existence of AIDS and the benefits of treatment. These uncertainties could lead women to stop treatment. These ideas were expressed by both the Spanish and sub-Saharan African women. As HIV is a “silent” disease, its nature leads to these kinds of doubts.

*“The other day I talked to a good friend of mine and I had a small argument with her because of the medication […]. She stopped taking the medication correctly: She split the pills in half. Because there are some people who say: ‘If this man weights 80 kg and I weight 40, why are they giving us the same doses?’ Then, I would have to take only half’. And a group of people were then taking only half of the medication.” (S1, Spanish)*

#### Facilitators

Women who did follow a regular course of treatment believed that it was beneficial for them.

*“It never crosses my mind to stop seeing the doctor; one goes to see the doctor to feel good, for the sake of health. If you don’t go to the doctor, you will be transmitting to others. (SA3, Equatorial Guinea)*

#### Immigrant women

In African participants, the doubt of the necessity to take a treatment was linked with the idea that AIDS could be a myth created because the “whites” were jealous of African fertility. The interviewee from Cameroon defined AIDS as the “Imaginary Syndrome to Discourage Lovers", which in French, matches with the disease acronym (*SIDA*). This statement was also identified in previous studies [12]. The possibility exists that traditional medicine will be a choice among HIV African patients.

*“In my country, if you have this disease, you can go to the traditional healers and he will give you very powerful herbs, some people said that they are better than the pills.” (SA1, Cameroon)*

### Psychological condition

In the moment that the women participants became aware of the infection, there were several psychological responses. Some of these helped to keep medical follow-up on track but others were a strong barrier.

#### Barriers

Denial of the disease appeared in our study as an obstacle to follow-up. The women said that going to the doctor and taking the treatment was a factor that continuously reminded them of the presence of the HIV.

The women who stopped treatment said that at that time, they did not want to think about the disease.

Sometimes, denial was expressed by saying: “It is better not to know anything”.

Denial can also be expressed through a strong desire to get pregnant after the diagnosis; some women would take the risk of getting pregnant without medical follow-up.

The emotional collapse can also be an obstacle for follow-up when patients become aware of the diagnosis in periods of intense mourning. In these situations, the psychic system falls apart and the person loses the ability to process information. This was the case of two women who learned they had HIV when their partners died of AIDS.

#### Facilitators

Nonetheless, we also detected mechanisms that encourage medical follow-up such as the expression of emotions to loved ones which strengthened links with the personal networks and enhanced their support.

*“Finally my boyfriend told me: ‘you are HIV positive’, I broke down….and with a million tears in my face, I told him: ‘yes’, and then he told me: ‘so now is when I have more reasons to stay by your side', and for me, that was…(sight) That got me out of the black hole where I was stuck, because I didn’t go out on the street, I didn’t talk to people, I didn’t eat. For me, the world was over, my mother was dead, my boyfriend was dead, I got this virus diagnosed and I had been told that it killed you.” (S4, Spanish)*

In order to avoid denial of the disease, women expressed the importance of believing in a transcendent reality: Something superior and benevolent that understands the reasons why the individual got the disease. These beliefs were expressed through faith in different religions, including animism.

*“I thank God, who gave me the disease and also de medication.” (SA8, Guinea Conakry)*

#### Immigrant women

Latin American woman similarly stated the importance they gave to praying or to participating in mystical ceremonies. African women expressed the importance of combining medical treatment with traditional remedies.

Among Spanish women, this kind of facilitator was not mentioned but a similar role was assigned to psychotherapists, who were perceived as professionals and who encouraged medical follow-up.

*“They saw I was very, very bad, they recommended me to go to a psychotherapist. And well, this woman has been helping me a lot, she encouraged me to start studying, doing things again, fulfilling my life again.” (S4, Spanish)*

The feeling of disorientation can be a reason for refusal to go to consultation: immigrants who knew they had HIV prior to their arrival in a new country explained that they needed to understand the cultural codes of their new country before going to medical consultation.

*“I didn’t go to my medical check-up for one year because I was starting in a new country, in French…" (LA2, Venezuela)*

The recent arrival in a country where cultural codes were unknown was a delicate stage in their lives and this could be detrimental for HIV care, treatment adherence and follow-up.

## Discussion

After a careful bibliographic search, we found that this was the first qualitative study to investigate the reasons HIV positive women terminate, or do not seek follow-up treatment in Spain. The research methods employed provide an explanation for the lack of follow-up treatment by immigrant women in an inductive way and from their own perspectives.

The factors that influence the cessation or continuation of follow-up treatment, as identified in our study were common among both immigrant and native women. The main difference was the increased vulnerability felt by immigrant women related to their social isolation, employment condition, socioeconomic stability and so on, which impacted their experience of having HIV. The women who had stopped medical follow-up and participated in this study started to attend again. This demonstrated how important it is to have supportive people who are aware of their HIV infection, to help them return to follow-up treatment. Nevertheless, one of the great difficulties in finding support is the stigma of HIV, which drastically reduces the social networks where patients can find support. For this reason, for most of the women, the doctor had become the only confidante aware of the disease.

## Doctor–patient relationship

We believe that in the healthcare field it is possible to implement simple measures that can improve the quality of doctor-patient interaction and lead to improved adherence to follow-up of HIV-infected immigrant women, without the need to invest large amounts of resources. Many solutions can be found with a better understanding of the barriers women face and by focusing on effective ways to help them overcome these obstacles.

The main findings of this study have been collected in an informative leaflet for health care professionals aimed at improving understanding of the difficulties that this population faces and to provide a guide on how to approach them. We illustrated them in the Table 2 and the leaflet is available online at: http://www.saludentreculturas.es.

**Table 2:** **Leaflet’s guidelines for healthcare professionals**

Leaflet’s guidelines for healthcare professionals	
**Doctor-Patient relationship**	- Use an accessible language for the patient according to their level of education, explain technical terms and ask open questions.
	- Use professional interpreters if the patient has no proficiency in Spanish. Avoid using family members, friends or untrained interpreters.
	- Explore the emotional sphere by asking questions such as: How are you feeling today? Do you think much about the disease? Try not to talk only about the physical aspect of the disease.
	- Ask if she feels comfortable talking about her sexuality with a male professional.
**Body - HIV relationship**	- Explain the stages of the disease and the advantages of a prompt treatment and follow-up to avoid these barriers.
**Employment**	- Find out the purpose of their immigration. If it is economic, remember that work may be a more important priority for the patient than health.
	- Bear in mind that the administrative status (documented/undocumented) and work conditions of the patient are fundamental to understanding if they will be able to attend follow-up consultations.
	- Be flexible with consulting hours, allow consultations by telephone and avoid making appointments for superfluous matters.
	- Verify if she has any family/friend who can help her to pick up the medication or can accompany her to the consultations.
**Gender roles**	- Ask if she has a partner or children (how many) and where they live.
	- If she is single, explore her personal desires, explain that a person with HIV can build a family.
	- Recommend strategies to make the use of condoms easier with their stable partner until they are ready to disclose their diagnosis (they can say, for example, they need to use it due to a gynecological problem).
	- Teach them how to use the masculine and feminine condoms and lubricants.
	- Encourage the patient to participate in enjoyable activities.
**Representations of AIDS**	- Explore what the woman thinks about HIV (now and before being infected) and what her family, friends and other people around her think about the infection.
	- Make a clear distinction between HIV and prostitution or promiscuity. This will help to prevent feelings of guilt and shame.
	- Pay attention to the fears and myths related to HIV.
**Emotional support received**	- Investigate if she has a support person (family or friend); and the quality of the relationship with her partner.
	- Try to get a support person involved in her medical follow-up.
	- Encourage the patient to create a support network.
**Trust in Biomedical system**	- Bear in mind the possibility of looking for an “expert patient” who can assist with your patient: this would be a woman who has HIV and who comes from the same culture as the patient, so she could provide support and improve the patient’s trust in the healthcare system.
	- The patient’s perceptions can also be a useful tool in this respect.
**Psychological condition**	- Separate the HIV diagnosis from death and from stigma.
	- Investigate the patients’ representations of the disease and the impact of the loss of people close to them (i.e. a child or a partner).
	- Explain the advantages of seeing a psychologist or psychiatrist when necessary and encourage them to seek support from traditional/religious practices, as long as they do not have harmful effects on the treatment.

Multidisciplinary teams can be especially useful in the first stages, as they promote a closer link to healthcare staff and encourage good relationships between the patient and her caretakers. To better understand the socio-economic and psychological reality of patients, these teams should be made up of doctors, nurses, psychologists, social workers and professional interpreters, in case the patient does not speak the provider’s language. The presence of a professional interpreter within a healthcare service is instrumental to creating a good relationship with the healthcare staff. Translations provided by friends or acquaintances are not recommend because they can seriously damage communication between patients and doctors and put confidentiality at risk [24].

### HIV-body relationship

The silent nature of HIV, due to the long asymptomatic stage of the infection, can act as an important barrier to follow-up. It also encourages the denial of the disease and leads to a lack of trust in the medical system. In this sense, we believe that it is important to understand the somatization in patients as a real effort to give the disease a body, a representation. These somatizations can also make healthcare professionals aware of the need to explain the stages of the disease and the benefits of early treatment. Furthermore, the healthcare models of the countries of origin (Latin America and sub-Saharan Africa) follow mostly a palliative model (people go to the doctor when they feel ill) [25]. This makes it even more difficult to convince patients of the need for follow-up in the asymptomatic stage.

### Employment

The difficult interaction between the working environment and the rigid structure of the healthcare system creates additional barriers for working women, the most vulnerable being those who have immigrated for economic reasons. Immigrant women usually have jobs sometimes called the 3Ds (“Dirty, Dangerous and Demanding”) [26], which do not leave much time for taking care of their own health. The lack of flexibility and the fear of loosing their jobs are very strong barriers to medical follow-up.

### Gender roles

One crucial moment when women had more problems adhering to treatment was when they were looking forward to finding a partner and creating a family (especially among women with a traditional conception of gender roles). Exploring the family context and the personal desires on the first sessions could help the women to know the possibilities for them of building a family.

### Social representations of AIDS

Representations of AIDS depend on the social environment and can affect the follow-up of patients. We observed cases of women who stopped coming to medical consultation because they did considered themselves as individuals at risk for HIV. Exploring what the patients think about HIV and what her family and relatives around her think about the infection could be an opportunity to give adequate information and avoid fears and myths related to this chronic disease.

For the immigrant women a clear distinction between HIV and prostitution or promiscuity could be necessary and could also prevent feelings of guilt and shame.

### Emotional support received

Furthermore, it is necessary to encourage women to find support outside of the healthcare team, as the study results show this is crucial to continued adherence to follow-up visits. This support does not necessarily to come from the family. The most important factor is that the person offering support is in a position of not judging the patient. For women who stopped taking their medication and who do not have any support, the referral to patient associations can be an interesting alternative. Creating a space in the healthcare centre for patient associations would allow contact with some of the most isolated women. Hospitals can also utilize “expert patients” or “navigator patients” [27] to serve as a link between the hospital world and the immigrant women. Studies on the impact of these figures could be a key to improving follow up visits of HIV immigrant women.

### Trust in biomedical system

As reported in other studies [15], the possibility the possibility to use traditional medicine to treat HIV without informing the healthcare professionals may occur.

Exploring the impression that women have about the medical treatment as well as other options they may consider (such as traditional medicine) would be advisable.

### Psychological condition

Finally, patients expressed a need to integrate HIV into their personal stories to make future plans again and to help them thrive. The native women valued the role of the therapist in helping them to “live a fulfilling life again”, as one of the interviewees stated. In the case of immigrant women, the role of the therapist is not so well-known and they can sometimes be suspicious. Instead they may prefer other kinds of support people, in accordance with their beliefs, such as a religious leader or a traditional healer. In the first medical consultations with patients, it is important to establish if there is such a support figure, and to clarify his/her role in improving the well-being of patients.

### Limitations

One of the main limitations in this study comes from the specific populations that were studied and the environment where it was conducted. Latin American and sub-Saharan immigrants, had access to a healthcare system with free coverage for HIV infection at the time of the study. In situations where immigrants come from other regions, such as Eastern Europe or Asia, the study results may be different. For those cases where access to healthcare services is impossible and/or the treatment is not free or affordable, additional barriers to follow-up treatment are obvious. This is a problem that may be aggravated in our own country if the restrictions affecting undocumented immigrants take effect [20].

Another limitation found in the study: the fear of discrimination. To avoid disclosure of the diagnosis, women tried to control the information. Some women refused to participate in the study because they wanted to maintain secrecy. Additionally, five women, four originally from sub-Saharan Africa, refused permission to record the interview due to the fear of their identity being revealed through the audios.

The difficult task of recruiting HIV positive immigrant women who stopped medical treatment led us to think that other barriers may exist. The economic crisis and the new movement of immigrants in order to find a job might be a reason for not being able to contact them and could also become a new barrier for HIV medical follow-up.

## Conclusion

The immigrant experience is a reality of the HIV pandemic in Western countries, where immigrant women are an especially vulnerable group. Barriers and facilitators to medical follow-up of immigrant women are very similar to the ones found for native women in our study. Nevertheless, these pitfalls and opportunities have a much greater impact on the former, especially when they serve as obstacles. To understand these barriers and how to deal with them is crucial for healthcare professionals to provide quality care tailored to the socio-cultural characteristics of patients. The main results of this study have been collected in a brochure available online at: http://www.saludentreculturas.es. This brochure is a new tool for healthcare professionals working in Spain with HIV positive immigrant women. Its objective is to help identify the main potential barriers for women immigrants to adhere to medical follow-up, and to transform these barriers into facilitators.

## References

[1] *UNAIDS Report on the Global AIDS Epidemic*. 2010. Available at: http://www.unaids.org/globalreport/. Date last accessed: Octuber 27, 2014

[2] Lang DL, Salazar LF, Wingood GM, DiClemente RJ, Mikhail I (2007). Associations between recent gender-based violence and pregnancy, sexually transmitted infections, condom use practices, and negotiation of sexual practices among HIV-positive women. J Acquir Immune Defic Syndr.

[3] Stirling M, Rees H, Kasedde S, Hankins C (2008). Introduction: Addressing the vulnerability of young women and girls to stop the HIV epidemic in southern Africa. AIDS.

[4] Collazos J, Asensi V, Carton JA (2007). Sex differences in the clinical, immunological and virological parameters of HIV-infected patients treated with HAART. Aids.

[5] Finkel DG, John G, Holland B, Slim J, Smith SM (2003). Women have a greater immunological response to effective virological HIV-1 therapy. Aids.

[6] Garcia de la Hera M, Ferreros I, del Amo J, Pérez Hoyos S, Muga R, del Romero J, Guerrero R, Hernández-Aguado I, GEMES (2004). Gender differences in progression to AIDS and death from HIV seroconversion in a cohort of injecting drug users from 1986 to 2001. J Epidemiol Community Health.

[7] Nicastri E, Leone S, Angeletti C, Palmisano L, Sarmati L, Chiesi A, Geraci A, Vella S, Narciso P, Corpolongo A, Andreoni M (2007). Sex issues in HIV-1-infected persons during highly active antiretroviral therapy: a systematic review. J Antimicrob Chemother.

[8] Prins M, Meyer L, Hessol NA (2005). Sex and the course of HIV infection in the pre- and highly active antiretroviral therapy eras. Aids.

[9] Currier J, Averitt Bridge D, Hagins D, Zorrilla CD, Feinberg J, Ryan R, Falcon R, Tennenberg A, Mrus J, Squires K, GRACE (Gender, Race, And Clinical Experience) Study Group (2010). Sex-based outcomes of darunavir-ritonavir therapy: a single-group trial. Ann Intern Med.

[10] Squires KE, Johnson M, Yang R, Uy J, Sheppard L, Absalon J, McGrath D (2011). Comparative gender analysis of the efficacy and safety of atazanavir/ritonavir and lopinavir/ritonavir at 96 weeks in the CASTLE study. J Antimicrob Chemother.

[11] Staehelin C, Keiser O, Calmy A, Weber R, Elzi L, Cavassini M, Schmid P, Bernasconi E, Furrer H, Swiss HIV Cohort Study (2012). Longer term clinical and virological outcome of Sub-saharan African participants on antiretroviral treatment in the Swiss HIV Cohort Study. J Acquir Immune Defic Syndr.

[12] Navaza B, Guionnet A, Navarro M, Estevez L, Perez-Molina JA, Lopez-Velez R (2012). Reluctance to do blood testing limits HIV diagnosis and appropriate health care of sub-Saharan African migrants living in Spain. AIDS Behav.

[13] Burns FM, Imrie JY, Nazroo J, Johnson AM, Fenton KA (2007). Why the(y) wait? Key informant understandings of factors contributing to late presentation and poor utilization of HIV health and social care services by African migrants in Britain. AIDS Care.

[14] Dang BN, Giordano TP, Kim JH (2012). Sociocultural and structural barriers to care among undocumented Latino immigrants with HIV infection. J Immigr Minor Health.

[15] Thomas F, Aggleton P, Anderson J (2010). ‘Experts’, ‘partners’ and ‘fools’: exploring agency in HIV treatment seeking among African migrants in London. Soc Sci Med.

[16] Ndirangu EW, Evans C (2009). Experiences of African immigrant women living with HIV in the U.K.: implications for health professionals. J Immigr Minor Health.

[17] Bandyopadhyay M, Thomas J (2002). Women migrant workers' vulnerability to HIV infection in Hong Kong. AIDS Care.

[18] Vigilancia epidemiológica del VIH en España (2012). Valoración de la epidemia a partir de los sistemas de notificación de casos de las CCAA.

[19] Perez-Molina JA, Mora Rillo M, Suarez-Lozano I, Casado-Osorio JL, Teira Cobo R, Rivas Gonzalez P, Pedrol Clotet E, Hernando-Jerez A, Domingo P, Barquilla Diaz E, Esteban H, Gonzalez-Garcia J (2012). Response to combined antiretroviral therapy according to gender and origin in a cohort of naive HIV-infected patients: GESIDA-5808 study. HIV Clin Trials.

[20] Perez-Molina JA, Pulido OF (2012). Assessment of the impact of the new health legislation on illegal immigrants in Spain: the case of human immunodeficiency virus infection. Enferm Infecc Microbiol Clin.

[21] Glaser B (1978). Theoretical Sensitivity.

[22] Strauss A (1987). Qualitative Analysis for Social Scientists.

[23] Gale NK, Heath G, Cameron E, Rashid S, Redwood S (2013). Using the framework method for the analysis of qualitative data in multi-disciplinary health research. BMC Med Res Methodol.

[24] Flores G (2005). The impact of medical interpreter services on the quality of health care: a systematic review. Med Care Res Rev.

[25] Inmigración, salud y servicios sanitarios (2013). La perspectiva de la población inmigrante.

[26] Employment and working conditions of migrant workers-Spain (2007). European foundation for the Improvment of Living and Working Conditions.

[27] Freund KM, Battaglia TA, Calhoun E, Dudley DJ, Fiscella K, Paskett E, Raich PC, Roetzheim RG, Patient Navigation Research Program Group (2008). National cancer institute patient navigation research program: methods, protocol, and measures. Cancer.

[CR28] The pre-publication history for this paper can be accessed here:http://www.biomedcentral.com/1471-2458/14/1115/prepub

